# Exploring the experiences of community first responders working in a UK ambulance service

**DOI:** 10.29045/14784726.2023.3.7.4.8

**Published:** 2023-03-01

**Authors:** Kelly Hird, Cliff Richardson

**Affiliations:** Yorkshire Ambulance Service NHS Trust; University of Manchester

**Keywords:** ambulance services, community first responders, qualitative research

## Abstract

**Background:**

Community first responders (CFRs) work voluntarily to support UK ambulance services by responding to emergencies. They are dispatched via the local 999 call centre and details of incidents in their local area are sent to their mobile phone. They have emergency equipment with them, including a defibrillator and oxygen, and attend a range of incidents, including cardiac arrests. Previous research has looked at the impact the CFR role has had on patient survival, but there is no previous research looking at the experiences of the CFRs while working in a UK ambulance service.

**Method:**

This study involved 10 semi-structured interviews, which took place in November and December 2018. One researcher interviewed all the CFRs using a pre-defined interview schedule. The findings of the study were analysed using thematic analysis.

**Results:**

The main themes from the study are ‘relationships’ and ‘systems’. The sub-themes of relationships are the relationship between CFRs; the relationship between CFRs and ambulance service staff; and the relationship between CFRs and patients. The sub-themes of systems are call allocation; technology; and reflection and support.

**Conclusions:**

CFRs support one another and are encouraging with new starting members. Their relationships with ambulance service staff have improved since CFRs first became active, but there is still room for improvement. The calls that CFRs attend are not always within their scope of practice, but the rate of this occurring is unclear. CFRs are frustrated with the level of technology involved in their role and feel it impacts them attending incidents quickly. CFRs reported attending cardiac arrests on a regular basis and the support that they receive afterwards. Further research should use a survey approach to further explore the experiences of the CFRs based on the themes raised in this study. Using this methodology would identify if these themes are unique to the one ambulance service where this was conducted, or if they are relevant to all UK CFRs.

## Introduction

A community first responder (CFR) formally volunteers in an ambulance service to attend 999 emergencies in their local area. They are in situ in both urban and rural communities across all UK ambulance trusts, and are trained in basic first aid, cardiopulmonary resuscitation and the use of an automated external defibrillator (AED). Some ambulance trusts have CFRs who administer drugs as well as medical gases depending upon their level of training. Other ambulance services have registered professionals with a clinical background volunteering for them. The National Association of Community First Responders is the national body for CFRs. However, their regulation depends upon individual ambulance service requirements.

Ambulance trusts across the United Kingdom have different criteria for what they will dispatch their CFRs to. However, all trusts will dispatch CFRs to cardiac arrests if they are on call and are the nearest resource. The research around the impact of CFRs at cardiac arrests is increasing. However, there is little research discussing the experiences of CFRs working in UK ambulance services more generally. Colquhoun et al. (2008) applied quantitative methods to investigate AEDs, comparing the static AED with the mobile CFR, and the impact on survival rates. The conclusion was that the static AED is a more effective strategy than the mobile CFR with an AED. In contrast, it is well documented that survival rates of patients are reduced, the greater the length of time from cardiac arrest to defibrillation (Nolan et al., 2006); hence, this is not a direct comparator.

The only comparator for CFRs is other ambulance clinicians. The gap in research into the experiences of CFRs while undertaking this vital work is stark and requires addressing with urgency due to the well-documented and published evidence concerning paramedics. Research involving paramedics has increased significantly in recent years, and varies from mental health problems associated with the role (Petrie et al., 2018) to the demands associated with shift work (Sofianopoulos et al., 2012). Mind (2016) published a survey of ambulance staff mental health in which 91% (n = 1352) of respondents said they had experienced low mood, stress or poor mental health while working in the ambulance service. If this previous research into ambulance staff is transferable to CFRs, there is a great need to address the support available for the volunteer staff.

## Methods

In order to explore the experiences of CFRs working in UK ambulance services, a qualitative approach was taken, as the research in this area is limited. This study sought and was given University Research Ethics Committee approval in order to protect participants. The study site is a UK ambulance service in the north of England, where the CFRs receive a weekly newsletter from the host organisation. An invitation to take part in the study was placed here, including the participant information leaflet; this detailed the purpose of the research and offered CFRs the opportunity to take part. Participants were advised in the advert to contact the researcher directly if they would like to take part in an interview. Participants were randomly allocated an identification number, to maintain anonymity, with the use of a randomisation table (https://www.randomizer.org/). Ensuring anonymity and allowing participants the time to consider whether they want to participate in the research conforms to the principles of informed consent. The interviews took place in November and December 2018.

The thematic analysis for this study followed the approach described by Braun and Clarke (2006) in their guidance for conducting thematic analysis. This methodology allowed the researcher to gain an in-depth understanding of the experiences and perspectives of the participants. The initial phase for thematic analysis was to become familiar with the raw data. The recordings were transcribed using true verbatim into a Word document. Included in the transcripts were the pauses and other non-verbal language (e.g. laughs and ‘hmm . . .’) to ensure that the transcripts were of the most value after completion, as the audio recordings were only available for a limited time. The transcription notes were in the form of a two-columned table, with the transcription in column one.

The second phase of the data analysis was the generation of initial codes from the transcriptions, some of which became apparent during transcription and the interview phase. There were no pre-set codes and so the codes were determined inductively from the data. The same strategy for determining which data belong in the coding system was applied to the whole dataset for transparency. This was the phase of idea development about what is taking place within the data (Savage, 2000). These codes were documented within column two of the table containing the transcriptions, allowing for comparison with other transcripts. Although this may be viewed as a reductionist view to data analysis (Clarke & Braun, 2013), it allowed for conceptualisation of the data. From these codes, basic themes were built to provide an overarching main theme from the data itself (Attride-Stirling, 2001). Themes were identified in the data through repeated meanings within the transcripts and thick description provided, as this method is most suited when investigating an under-researched area (Braun & Clarke, 2006).

Finally, the raw data were reviewed again to ensure that the themes sufficiently covered all aspects of the data. It was at this point that adaptations were made to themes, to ensure that they were relevant to the data. Large themes were reduced to form sub-themes. These sub-themes were linked to the wider theme but warrant a section for discussion, with supporting quotations from the raw data. The naming of the themes took place at this point based upon the data in each of them.

## Results

In line with the aims of this study, 10 semi-structured interviews were completed for this study. The response rate to the advert in the weekly newsletter was encouraging: 32 CFRs responded wanting to take part in an interview, within the first three days. Such a high response rate was not anticipated, and the sampling strategy in the protocol was to interview the first 10 respondents, and so this was adhered to.

The participants had an age range of 29 to 66 years (mean 50.9 years), and length of CFR service varied from a few months to more than 10 years (mean 4.6 years). Two CFRs were identified as working rurally, two urban and six said they volunteered in both. All interviews took place in a pre-booked private room in an ambulance station local to the CFR, lasting between 29 and 78 minutes (mean 45 minutes).

### Theme 1: relationships

Relationships are key to the role and to the work the CFRs undertake. The CFRs reported three main relationships in their role: with one another; with salaried ambulance service personnel; and with patients.

#### Sub-theme 1: relationship between community first responders

The relationship between CFRs appeared to be mostly professional and supportive. CFRs who were already active in the role encouraged others to join the scheme, as well as welcoming new members when they joined. This appeared to continue when meeting up with other groups of CFRs for training and meetings:

*We encourage people to join. I mean, we have three new members, so everyone is coming up to my house next week to have coffee, meet the new team and just get to know each other.* (CFR6)

*We have our two-monthly meet-up. We have a meet-up with other schemes from other areas, we all get on well*. (CFR7)

Although there are varying reasons for joining the CFR scheme, the CFRs appeared to see themselves as a self-managed, self-contained unit. The more experienced CFRs would attend incidents with new starters to help them with their confidence in the role, and termed this ‘buddying up’ or ‘piggy backing’:

*It actually came through as a fall, and it wasn’t. Fortunately, on that occasion there was one of my colleagues with me because he was less confident and asked if he could piggy back my shift . . . We’ve now got two younger CFRs that have joined, I try to help them wherever I can*. (CFR5)

*It’s just getting hands on with your patient and more exposure. You get to that, either with the buddy system with other CFRs or just chatting to the crews on the road*. (CFR3)

There were instances where CFRs highlighted concerns over the types of people applying for the role of CFR. CFRs raised concerns over recruitment strategies and a lack of trust in some of their colleagues:

*I think sometimes that people are attracted to the CFR role who think that they are paramedics and they scare me. They are not weeded out very quick but we don’t want people to be over-reaching themselves*. (CFR1)

*Part of the recruitment wasn’t picking personalities, unfortunately like any job, you do need to have a certain level of skill, and unfortunately we were tending to pick people just because they wanted to volunteer, rather than picking them as they were suitable to do the task*. (CFR9)

#### Sub-theme 2: relationship between community first responders and ambulance service staff

CFRs have been active in the host trust for over 10 years, and the relationship between CFRs and ambulance service staff appears to have changed over time. The initial feelings from the longer-serving CFRs indicated that they were treated with negativity and fear. This has, in most part, turned to a professional, respectful relationship:

*They were strangely fearful that we were there to replace them. Initially, I’m going back a long time, crews were not happy in many instances, others were good and very welcoming, encouraged us to observe and learn skills*. (CFR1)

*The crew arrived, I went out to offer them the PRF, the crew were just . . . one of them in particular was just nasty. It nearly put me off wanting to do it again. [He said] ‘I don’t know why they do it, why do we need you?’ It was in front of the patient at the time, I just stood there. Fortunately they are few and far between now but unfortunately in the early days it was quite a regular occurrence.* (CFR9)

The reported mutual respect between CFRs and ambulance service staff was widespread, with the exception of some individuals from the ambulance service who continued to foster negative relationships. CFRs reported that there were still isolated incidents of this occurring when attending to a scene where a CFR and a salaried ambulance staff member were present:

*I’d used all my oxygen and we have to get a replacement oxygen, and she was downright rude. I was like ‘can I replace this oxygen?’ and she was horrible to me. So, I said thank you very much for the oxygen and she just slammed the ambulance door on me.* (CFR4)

Although crew attitudes vary, CFRs were quick to justify the inappropriate behaviour of crews. After discussing ambulance staff behaving in a negative manner to them, they then appeared to make excuses for this behaviour:

*I haven’t had a bad experience on the road at all. They have all been fully supporting, one or two have been a little bit short, whether that’s in their nature or because they have had a pretty shabby day or night, I don’t know and I don’t enquire.* (CFR3)

*There have been a couple of occasions where crews have taken over from you and they might be a bit blunt with you or ‘ok we don’t need you’, ‘Right ok, see you later’. But again, you don’t really know where that crew have been and dealt with previous to you, they might have been to a nasty accident beforehand.* (CFR8)

#### Sub-theme 3: relationship between community first responders and patients

CFRs were keen to discuss how they provided reassurance to patients. The CFRs’ perceived route to achieving this goal was to calm the patient down prior to the ambulance crews arriving. Many disclosed that the presence of an ambulance response was intervention enough:

*A lot of time it’s TLC and I’m not a paramedic, I don’t profess to be but you do calm people down just by walking in the room.* (CFR2)

*We could go and help, erm, even if that’s just comfort, cos quite a few of my patients have said that they feel much better just because I’ve arrived. I’m very conscious that I’m not clinical, but just to have that calming effect because we have got the ambulance service logo on*. (CFR3)

Although the public may not fully understand the workings of the ambulance service, they seemed grateful for the attendance of someone who represents the ambulance service, rather than noting that the responder may not be a paramedic. This was reflected in many of the interviews with the CFRs:

*You walk in through the door, ‘Hi, I’m from the ambulance service’; and everyone just goes ‘brilliant, great’ . . .* (CFR7)

*It makes me laugh when they say, ‘the paramedic is here’, I’m like ‘no, I’m not, I’m a CFR’. But they all say thank you.* (CFR2)

### Theme 2: systems

CFRs fit within an ambulance service in many different roles, and the complexity of this is reflected within the theme of systems.

#### Sub-theme 1: call allocation

Part of the role of the emergency operations centre (EOC; 999 call centre) staff is to ensure that CFRs are only sent to patients who meet the advanced medical priority dispatch system (AMPDS) code criteria that is deemed suitable. This is a pre-defined list of incidents that CFRs can attend and excludes some incidents, including those where there is deemed to be a risk, for example involving alcohol or potential violence. There was a general feeling among CFRs that this was not always common practice, and that details were either coded inaccurately or they were allocated to incidents incorrectly:

*You may be aware that as CFRs there are lots of things we don’t go to. We don’t go to fallers normally, suicides, we don’t go to people under the influence of drugs or alcohol. Of course, that is all based on what comes through to control (EOC) and their understanding. So, I’ve been to suicides, people under the influence of drugs and alcohol*. (CFR1)

*There’s been a couple of call-outs that I shouldn’t have been called to. One was a lady with mental health issues who called and said she’s had a fit, but it turned out she’d taken an overdose of Viagra*. (CFR4)

Further to this, there were some extreme examples of CFRs attending incidents that were questionable in terms of the data shared with the call handler in the EOC:

*I have had some strange ones, I have had one that came in as a murder . . . hmm . . . which I have to say was a very strange experience, you know, when you’re suddenly having to give all your details to the police cos you were the first one there*. (CFR9)

*I had another experience which wasn’t so good. I had two actually . . . One was, it turned out to be a major trauma, I know we don’t normally go to major trauma, but it was in the middle of the night, about 2 a.m*. (CFR5)

#### Sub-theme 2: technology

CFRs are mobilised using a mobile phone, issued by the host trust, via a call from EOC staff. They then have to locate the address to attend the call. Common practice for this appears to be that CFRs put the address/postcode in their satellite navigation within their own personal vehicle, taking valuable seconds and potentially delaying activation times. They found this process cumbersome and were keen to state the issues with the allocated technology:

*I went to someone who was choking, and they were properly choking, and it was a successful outcome, don’t get me wrong. I was first on scene, but I was livid about how long it took me to get mobilised.* (CFR3)

*The phones are very old in style and because we are rural you can quite often find that you have no signal. So, you arrive, and you have to remember what time you have arrived because you have no signal, so you send a text and it simply doesn’t go, so an update on the phones would be very nice*. (CFR6)

This lack of robust technology added frustration to both the CFRs and EOC when sharing details of incidents attended:

*So sometimes you can get quite a terse response from some of them (EOC) if you don’t quote your job number exactly and our job number is on the phone, but if you’re using the phone you can’t do it cos it knocks off the phone on some of them.* (CFR9)

#### Sub-theme 3: reflection and support

During the interviews, participants spoke candidly about the patients they had attended and how it had made them feel. The participants were open about the number of cardiac arrests that they had attended. In some cases, the number was high:

*Ours is a busy scheme and over the past seven years I’ve attended 30-odd cardiac arrests.* (CFR9)

*I’ve done 17 cardiac arrests in two and a half years. I did seven in the space of four weeks in the summer.* (CFR10)

The participants also reflected on the level of support received after attending a traumatic job or a cardiac arrest with varying degrees of satisfaction. The CFRs reported that the support mechanisms are in place and extremely successful in some areas:

*We get good support from our area CFR. XXX, she’s really good. She’s always ringing us and asking if we are alright. The call centre, when we clear a call, they always ring us up and ask if we are alright.* (CFR2)

*My trainer was very supportive and followed up to see why I had been dispatched . . . officially I should have been stood down. Again, no problems with doing that. So the welfare has far exceeded my expectatio*n. (CFR5)

However, this level of support was not reported by all CFRs who took part in the interviews. Other CFRs reported a lack of support, and identified the cause of this to be when there was a change of trainer in the area:

*So, one person trains us to do something and that’s meant to be standard procedure and then the other trainer will come and say, ‘that’s all wrong’. And so we’re all confused.* (CFR10)

*We’ve just swapped our trainer, our old trainer had come back again, the one we had in between, we didn’t get much practice training. So, the only time we saw them was for our re-quals.* (CFR4)

## Discussion

As CFRs are a branch of the ambulance service volunteer sector, they have links with many key stakeholders: other CFRs, ambulances crews and members of the public, either as patients or family members. These relationships build the beginnings of understanding the experiences of the CFR in their role. The reported relationships between CFRs seemed to vary between participants, with most being positive but some expressing mistrust. However, they appeared to support one another and form peer relationships. There was specific mention as to how they support new volunteers and welcome them into their schemes, and ‘buddying up’ was common. This is something that the longer-serving members appear to have taken upon themselves to organise, as the formal introduction to other members into their local scheme may not occur straightaway. Hager and Brudney (2004) suggest that this welcoming behaviour by already volunteering personnel aids with the retention of new volunteers.

The relationship between CFRs and ambulance service staff was something which longer-serving CFRs suggested has changed over time. The CFRs expressed that although there can be negativity from ambulance crews, this is not as commonplace as it was several years ago. Previous literature suggests that the perception of salaried staff in other roles towards volunteers highlights negative connotations due to the fear of job losses (Mallum, 2017). The longer-serving CFRs were keen to discuss how behaviour towards them from ambulance staff had improved since the volunteer role began, and specifically mentioned ‘job losses’ as a fear factor in the early days of CFRs. This is in line with previous research from Gaskin (2003), who also found that salaried staff can be suspicious and question the motivations of volunteers.

The CFRs reported that members of the public did not appear to fully understand their role, which is not unexpected as it is well documented that the ambulance service is misunderstood and inappropriately utilised on a wider level (Booker et al., 2014). The CFRs who took part in this study felt that the patients were calmer with them in attendance, and their level of clinical knowledge was not a factor.

The type of incidents that CFRs attend is a pre-defined list held in the EOC and directly related to the AMPDS code allocated to the call. However, participants who took part in these interviews acknowledged that this was not always the case and that they had been allocated to calls which are outside their scope of practice. This is congruent with previous research which suggests that the precision of call allocation is largely under-investigated (Snooks et al., 2002), suggesting that there may be disparity between the call and what is actually on scene when an ambulance service response arrives. CFRs were therefore attending incidents involving drugs, alcohol and potential violence; the impact of this on the CFR is unclear.

Technology was also a concern. CFRs appear to be increasingly frustrated at the lack of investment in what they consider a basic requirement for the role: a device to alert them to incidents they can attend. This frustration continued when CFRs were trying to contact the EOC, as EOC staff could be abrupt with them when calling afterwards with the incident details. Ensuring CFRs have the appropriate technology to carry out their role is vital for CFR schemes moving forwards.

During the interviews, the CFRs disclosed varying numbers of cardiac arrest attendances when discussing their role. In some instances, the number of cardiac arrests attended by CFRs was at least equal to what a paramedic would expect to attend per annum, although it is unclear if this is transferable to all CFRs. Previous research suggests that most non-specialist paramedics can expect to attend around two cardiac arrests per year (Dyson et al., 2015). The missing detail throughout this previous research is how many cardiac arrests CFRs are attending each year. CFRs disclosed varying levels of support following their attendance at a cardiac arrest, which they attributed to their local trainer.

### Further research

A larger study involving other ambulance services to quantify the claims made during these interviews, with a focus on relationships between salaried and non-salaried ambulance staff, would be valuable. Data should also be analysed to explore the CFRs’ claims of attending incidents outside their agreed scope of practice.

### Limitations

The study was only conducted in one UK ambulance service. Study sampling could have been more targeted had the response rate been anticipated, and participants could have been invited based on the area they worked in and years of experience. As the participants are members of the ambulance service, the interviewer being a paramedic could have inserted a level of bias into the study. This could be in the responses to relationships with other ambulance staff or the CFRs reflecting and justifying the inappropriate behaviour. The dependability element of maintaining trustworthiness for this study was lacking, as there was no formal audit check of the results by an external party, although no new codes were identified at the last interview and data saturation was achieved.

## Conclusions

This study was conducted in one UK ambulance service serving a population of around 5 million people over 6000 square miles, and as such the results should be treated with caution. CFRs are sufficiently motivated to undertake their role, but mechanisms are required to support them. Longer-serving CFRs appear to take on the role of a mentor to new CFRs and they support one another in a non-formalised manner through ad hoc peer groups. The relationship between CFRs and ambulance staff is mainly a very encouraging, nurturing environment but this has not always been the case.

CFRs do not complain when attending to incidents that are not in their scope of practice and carry out their role with pride. They face challenges with technology, and the delays were a source of frustration as they would like to get to patients more efficiently.

## Author contributions

KH conceived the idea, conducted the research and wrote the manuscript. CR provided overall supervision and reviewed the manuscript. KH acts as the guarantor for this article.

## Conflict of interest

None declared.

## Ethics

University of Manchester Research Ethics Committee approval (REC Ref:2018-4755-7125).

## Funding

This article received an NIHR grant to complete an MRes in Clinical Research (MRES-2015-03-018-305).
